# Editorial: Bioinspired Design and Control of Robots With Intrinsic Compliance

**DOI:** 10.3389/fnbot.2020.00064

**Published:** 2020-09-17

**Authors:** Yongping Pan, Zhao Guo, Dongbing Gu

**Affiliations:** ^1^School of Data and Computer Science, Sun Yat-sen University, Guangzhou, China; ^2^School of Power and Mechanical Engineering, Wuhan University, Wuhan, China; ^3^Wuhan University Shenzhen Research Institute, Shenzhen, China; ^4^School of Computer Science and Electronic Engineering, University of Essex, Colchester, United Kingdom

**Keywords:** series elastic actuators, variable stiffness actuators, human-robot interaction, compliant control, wearable robots, walking robots

Intrinsic compliance, i.e., passive compliance, is one of the crucial properties of human and biological systems. In mammals, compliance results from the viscoelastic properties of muscle fibers and the series-elastic tendon structures which can be modulated at the muscle and joint level through the activation of the agonist and/or antagonistic muscles. Several technologies have been proposed to mimic the intrinsic compliance, such as series elastic actuators (SEAs) with fixed compliance, variable stiffness actuators (VSAs), and soft artificial muscles. There is an ever-increasing interest in implementing robots with intrinsic compliance to the fields of e.g., wearable robotics and walking robotics, because of their ability to absorb impact shocks, to safely interact with users, and to store and release energy in passive elastic elements.

One critical barrier to the development of robots with intrinsic compliance is the necessity for greater design inspiration and integration from bionic viewpoints. For instance, the design of compliant actuators to mimic the real muscle function is difficult because of the complex muscle structure and biomechanical properties. Besides, the control of robots with intrinsic compliance is still challenging due to the complexity and modeling difficulty of compliant components. For instance, the physical coupling between stiffness and position mechanisms in VSAs makes the control design complicated. How to control robots with intrinsic compliance in a more efficient way using bioinspired techniques in model learning, policy learning, and disturbance estimation, is an exciting topic.

This Research Topic is organized under the section “Bioinspired Design and Control of Robots with Intrinsic Compliance” within Frontiers in Neurorobotics. The collected articles are classified into three groups, where the first group focuses on robotics with fixed compliance, the second group focuses on robotics with variable compliance, and the third group include miscellaneous design and control methods which would be useful for robotics with intrinsic compliance.

In the first group of the articles, the article by Ma et al. proposes a proportional-derivative control method based on Extended Kalman filters for a robot arm with flexible joints under incomplete state feedback. Experimental results show that the proposed method is effective and has excellent trajectory tracking performance. In physical human-robot interaction, ankle joint muscle reflex control remains promising in human bipedal stance. The article by Cao et al. presents a specialized ankle joint muscle reflex control method for human upright standing push-recovery. The proposed method was implemented on a SEA-driven robot ankle joint, where the SEA has the potential to mimic human muscle-tendon unit. Experimental results indicate that the proposed method can easily realize upright standing push-recovery behavior that is similar to the original human behavior. The article by Zhang et al. focuses on modeling and control of a cable-driven rotary SEA for an upper limb rehabilitation robot, where both torque and impedance controllers were developed for the robot. Experimental results show that the proposed method can achieve stable and friendly actuation over a long distance. The article by Ruppert and Badri-Spröwitz presents a robotic leg design with physical elastic elements in leg angle and virtual leg axis direction. It is shown that the robotic leg with a gastrocnemius inspired elasticity possesses elastic components deflecting in leg angle directions, and can store hip actuator energy in the series elastic element. The advantages of the mechanical design with respect to energy efficiency in locomotion is shown by a vertical drop experiment.

The second group of the articles is about the control of VSAs which is still challenging due to model perturbations such as parametric uncertainties and external disturbances. The article by Guo et al. focuses on modeling and control of VSAs for new-generation compliant robots, where a novel modeling method is applied to analysis the VSA dynamics, and a non-linear disturbance observer-based controller is proposed to control both stiffness and position of VSAs under model perturbations. Experimental results have verified the effectiveness of the proposed method. The article by Lukić et al. presents a cascade control structure for the simultaneous position and stiffness control of antagonistic tendon-driven VSAs. The proposed controller has the ability to accelerate, stabilize, and reduce oscillations, which are important in systems such as tendon-driven compliant actuators with elastic transmission.

The third group of the articles presents one robot control method and two robot design methods which would be useful for robotics with intrinsic compliance. Robot force control can enhance compliance and execution capabilities. However, it is challenging for redundant robots, especially when there exist risks of collisions. The article by Zhou et al. proposes a collision-free compliant control strategy based on recurrent neural networks to save unnecessary energy consumption. Numerical results validate the effectiveness of the proposed controller. Modern engineering problems require solutions with multiple functionalities to meet their practical needs for handling a variety of applications in different scenarios. Conventional design paradigms for single design purpose may not be able to satisfy this requirement. Tan et al. proposes a novel system-of-systems bioinspired design method framed in a solution-driven bioinspired design paradigm. Eight steps of the design process are elaborated and a case study of reconfigurable robots is provided in the article. The last article by Chang et al. presents a series of alternative uses of structural compliance for the development of simple, adaptive, and compliant underactuated robotic grippers that can execute a variety of manipulation tasks. The grippers employ mechanical adaptability to facilitate and simplify the efficient execution. The efficiency of the grippers is experimentally validated via three different types of tests.

## Author Contributions

All authors listed have made a substantial, direct and intellectual contribution to the work, and approved it for publication.

## Conflict of Interest

The authors declare that the research was conducted in the absence of any commercial or financial relationships that could be construed as a potential conflict of interest.

